# Optimization of implant selection and positioning for reverse total shoulder arthroplasty using three-dimensional computed tomography–guided simulation software with scapulothoracic motion

**DOI:** 10.1016/j.jsea.2026.100049

**Published:** 2026-06-30

**Authors:** Katelyn E. Parsons, Devika A. Shenoy, Samuel G. Lorentz, Eoghan T. Hurley, Alessandro Navacchia, Michael Moverman, Jay M. Levin, Christopher S. Klifto

**Affiliations:** a Department of Orthopaedic Surgery, Duke University School of Medicine, Durham, NC, USA; b Smith+Nephew, Pittsburgh, PA, USA

**Keywords:** Reverse total shoulder arthroplasty, 3D simulation, Glenohumeral motion, Scapulothoracic motion, Implant, Positioning

## Abstract

**Background:**

Although three-dimensional (3D) simulation has improved accuracy of implant placement, its role in optimizing postoperative range of motion (ROM) remains incompletely defined. The purpose of this study was to use a 3D simulation model incorporating scapulothoracic motion to evaluate how implant variables for reverse total shoulder arthroplasty (rTSA) impact standard ROM and functional activities of daily living.

**Methods:**

Patients undergoing rTSA from August 2025 to October 2025 were included. Demographics were recorded. Implant parameters recorded included glenosphere size, eccentricity, glenosphere lateralization, polyethylene insert thickness, humeral neck-shaft angle (NSA), humeral stem version, and baseplate version. Outcomes were total ROM, with secondary analyses assessing the effect of individual implant variables on ROM. A 3D simulation model (CORIOGRAPH MODELER, Smith+Nephew) with scapulothoracic and glenohumeral-simulated motion evaluated impingement-free ROM across all implant parameters and 12 motions.

**Results:**

Forty-nine patients were included at a median 70.1 years old. Most common surgical indications were glenohumeral osteoarthritis (34.6%) followed by rotator cuff arthropathy (26.5%). Across simulated configurations, larger glenospheres, eccentric glenosphere positioning, and increased glenosphere lateralization were associated with greater impingement-free ROM (*P* < .05). Higher NSA was associated with lower ROM (*P* < .05). Multivariable regression found NSA and glenosphere size to be the strongest independent contributors to maximal ROM across multiple motions.

**Conclusion:**

Using a 3D simulation model incorporating scapulothoracic motion, rTSA implant positioning was shown to influence impingement-free ROM. Larger and eccentric glenospheres with increased lateralization consistently improved ROM, while higher NSAs limited motion across multiple planes. NSA and glenosphere size were the strongest independent predictors of maximal ROM.

Reverse total shoulder arthroplasty (rTSA) is an option for surgical reconstruction of the shoulder for various pathologies, including rotator cuff arthropathy (RCA), severe glenoid bone loss, and revision arthroplasty.^2^^,^^14^ Making up the majority of primary shoulder arthroplasties, rTSA alters glenohumeral mechanics by switching glenoid and humeral articulation.^16^^,^^25^ While rTSA is beneficial in cases of a deficient rotator cuff, it is also associated with higher rates of early complications, including instability caused by insufficient soft tissue tension, and generally provides less restoration of range of motion (ROM) compared to anatomic total shoulder arthroplasty (aTSA).^23^^,^^24^

Postoperative ROM improves significantly compared with preoperative values for both aTSA and rTSA, with gains across all planes after aTSA and improvements limited to flexion and external rotation after rTSA.^12^^,^^32^^,^^35^ There are many variables that can impact postoperative ROM, most predictively being preoperative ROM followed by others such as pathology, patient factors such as sex and comorbidities, and variables in surgical technique.^13^^,^^17^^,^^22^^,^^26^^,^^27^^,^^37^ Specifically, implant positioning and design are critical determinants of postoperative ROM. While various principles are supported within medical literature, there is no clear consensus regarding optimal implant positioning that increases ROM while limiting complications.

Given the importance of surgical planning, three-dimensional (3D) simulation models based on patient-specific computed tomography (CT) imaging have gained interest as tools to optimize implant positioning. Prior studies demonstrate that 3D planning improves accuracy of implant placement compared to traditional two-dimensional imaging by allowing more precise measurements and more consistent surgical decision-making.^8^^,^^15^^,^^34^^,^^43^ However, medical literature on the use of 3D simulation to optimize ROM after shoulder arthroplasty remains limited, with available studies constrained by small sample sizes or narrow focus on standard anatomic planes, frequently overlooking the complex kinematics required for daily function.^5^^,^^18^^,^^29^^,^^40^^,^^42^ In addition, most available literature looks solely at glenohumeral motion and does not include scapulothoracic motion, restricting accuracy of simulation. Therefore, the purpose of this study was to use a novel 3D simulation model incorporating scapulothoracic motion to evaluate how systematic adjustments in implant variables for rTSA impact both standard ROM and functional activities of daily living (ADLs). The hypothesis was that larger glenosphere sizing and lateralization would result in higher ROM.

## Materials and methods

### Patient cohort overview

This retrospective cohort study included a consecutive series of patients who underwent rTSA between August 2025 and October 2025. Patients provided informed consent after thorough counseling on surgical risks and alternative treatment options. Following institutional review board approval, patient data were collected on preoperative factors, implant variables, and resultant ROM. Preoperative factors included age at surgery, sex, and surgical indication. Surgical indications in this cohort included RCA and glenohumeral osteoarthritis (GHOA).

### Computer three-dimensional simulation model

**Figure 1:**
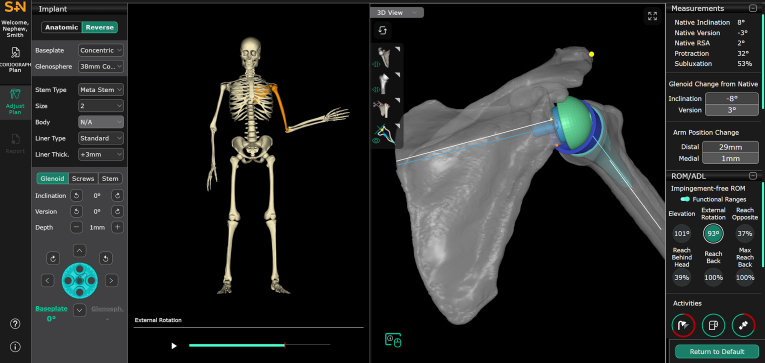
CORIOGRAPH MODELER preoperative planning software demonstrating the adducted rotation functional motion.

CORIOGRAPH MODELER (Smith+Nephew, Pittsburgh, PA, USA) was used to preoperatively plan each shoulder. It is a CT-based preoperative planning software that allows implant selection and placement based on the patient's bones reconstructed from CT images and simulates several ranges of motion including both scapulothoracic and glenohumeral kinematics (Fig. 1). Virtual plans were obtained for each patient using AETOS components (Smith+Nephew). Meta Stem size was selected to best match the patient's anatomy. A target of 80% of seating was used to plan baseplate position. Wedged baseplates were used to correct native inclination and retroversion deformities. Baseplate inferior inclination was targeted. The plans were conducted by preoperative planning engineers and reviewed and modified as needed by the senior author.

Implant variables adjusted for each patient included the following: glenosphere size (34 mm, 38 mm, 42 mm), concentric or eccentric glenosphere, glenosphere lateralization (0 mm, 3 mm), polyethylene insert thickness (0 mm, 6 mm, 15 mm), humeral neck-shaft angle (NSA; 125°, 135°, 145°, 155°), humeral stem version (10°, −10°, −30°), and baseplate retroversion (0°, 10°). A one-variable-at-a-time approach was used to isolate the effect of each implant parameter on ROM and impingement. A baseline configuration was established for each patient consisting of planned glenosphere size (determined based on native glenoid anatomy), 0 mm polyethylene liner thickness, 135° humeral NSA, −30° humeral stem version, and 0° baseplate version. When testing each parameter, only that variable was modified while all others remained at baseline settings. After evaluating each parameter, the component was returned to its baseline configuration before testing the next variable. This resulted in 19 possible implant configurations per patient.

**Figure 2:**
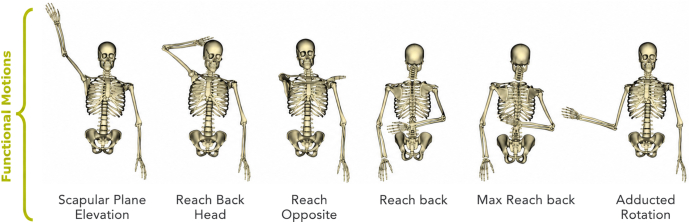
Diagram displaying the 6 functional planes of motion simulated in CORIOGRAPH.

CORIOGRAPH MODELER was then used to evaluate bone-on-bone and implant-on-bone impingement for the different plans using individual bone models and selected implant models. The following 12 motion parameters were assessed: 6 standard motions (flexion, extension, adduction, abduction, internal rotation, and external rotation), in which only the glenohumeral joint was moved about its primary axes, and 6 functional ADLs (elevation, adducted rotation, reach back head, reach opposite, reach back, and maximum reach back), which included both scapulothoracic and glenohumeral motions. The 6 functional motions were obtained with a full-body skeletal computer model (LifeMOD [Smith+Nephew, Pittsburgh, PA]), which tracked marker-based motion capture data collected on healthy adults (Fig. 2). The full-body computer model was validated comparing scapulothoracic and glenohumeral model kinematics to biplane fluoroscopy experimental data and following the American Society of Mechanical Engineers Verification and Validation 40 standard and the Food and Drug Administration Guidance for Computational Modeling and Simulation in Medical Device Submissions.^1^^,^^3^^,^^19^ The motions derived from the full-body simulations were applied within CORIOGRAPH MODELER to each of the individual preoperative plans. Personalized scapula posture was accounted for in these functional ADL impingement simulations. Specifically, the scapula position relative to the torso in the patient's CT scan was used as the starting point for the 6 functional simulations.

ROM values for standard and functional motions were recorded at the point of first impingement, and comparative analyses were performed to identify implant configurations that maximized ROM while minimizing impingement across motion domains. The ROM output for 4 of the 6 functional motions (reach back head, reach opposite, reach back, and maximum reach back) was expressed in percentage of completion rather than degrees because rotations in different planes were involved. Most common impingement point anatomically was recorded for each configuration per ROM parameter.

### Outcomes

The primary outcome of this study was overall shoulder ROM achieved across implant configurations. Secondary outcomes included the effect of individual implant design variables on ROM, specifically glenosphere size and lateralization, concentric versus eccentric glenosphere positioning, liner thickness, NSA, humeral stem version, and baseplate version.

### Statistical analysis

Demographic and perioperative variables were collected and descriptively analyzed across the entire cohort. Continuous variables were reported as median values with interquartile ranges, and categorical variables were reported as frequencies and percentages. Mean values for each ROM parameter were calculated for each implant variable setting in addition to mode impingement location. Multivariable linear regression was utilized to assess the relative contribution and strength of association of each individual implant parameter for achieving maximal ROM. Models controlled for age and results were reported as absolute standardized beta coefficients to reflect the magnitude of the effect of each implant parameter with the respective ROM value. *P* value <.05 was considered significant. All analyses were conducted in R (version 4.5.2; R Project for Statistical Computing, Vienna, Austria).

## Results

### Overall cohort details

There were 49 patients included who underwent a rTSA. The median age of the cohort was 70.1 years old (Q1: 66.3, Q3: 73.9) with 57% of the cohort being female. The most common surgical indication for rTSA was GHOA (n = 17), followed by RCA (n = 13), and concomitant GHOA with RCA (n = 12). Full details of the overall patient cohort can be found in Table I.

**Table I:** Demographics of the overall patient cohort.

Variable	Overall patient cohort (n = 49)
Age (years)∗	70.1 [66.3, 73.9]
Sex	
Female	28/49 (57.1%)
Male	21/49 (42.9%)
Surgical indication	
Rotator cuff arthropathy (RCA)	13/49 (26.5%)
Glenohumeral osteoarthritis (GHOA)	17/49 (34.7%)
Both RCA and GHOA	12/49 (24.5%)
Glenoid fracture	1/49 (2.0%)

*RCA*, rotator cuff arthropathy, *GHOA*, glenohumeral osteoarthritis.

∗ Median (Q1, Q3); n/N (%).

### Range of motion per implant design variable

Across the simulated implant configurations, larger glenosphere sizes and the use of eccentricity or lateralization led to improved ROM (Table II). Increasing glenosphere size from 34 mm to 42 mm improved nearly all motion parameters (eg, flexion 65.6° to 76.1°, internal rotation 43.8° to 65.2°, extension 83.6° to 98.8°, external rotation from 24.3° to 36.8°). The combination of a 42 mm glenosphere with +3 mm lateralization and eccentric positioning yielded the highest recorded values for nearly all standard motions: flexion 98.2°, adduction 27.6°, internal rotation 80.0°, extension 115.1°, and external rotation 59.3°. Similar trends were observed for functional ADLs, with the same configuration achieving the highest values for elevation (119.3°), reach opposite (49.9%), reach back head (52.8%), and maximum reach back (93.8%).

**Table II:** Average degrees for standard ROM and functional ROM by implant variable.

Variable	Standard ROM∗						Functional ROM∗					
	Flexion	Adduction	Internal rotation	Extension	Abduction	External rotation	Elevation	Adducted rotation	Reach opposite	Reach back head	Reach back	Max reach back
Glenosphere size (mm)												
34	65.6	9.5	43.8	83.6	64.6	24.3	101.3	60.9	41.7	41.5	79.3	75.1
38	62.8	10.2	46.4	71.4	57.0	26.1	95.5	63.3	37.8	41.4	76.4	74.3
42	76.1	14.3	65.2	98.8	67.1	36.8	97.2	69.6	39.5	44.8	91.5	88.6
38 + 3	76.2	13.2	49.5	88.7	66.9	34.4	104.3	68.2	44.7	45.0	84.5	84.9
42 + 3	86.6	18.1	64.5	105.0	71.0	44.2	106.4	75.4	46.9	48.9	48.9	93.7
38 eccentric	82.9	21.1	71.5	101.9	78.7	47.1	112.0	75.7	41.8	45.7	88.9	89.2
42 eccentric	87.1	22.9	76.0	109.0	78.0	52.5	110.5	79.7	43.8	48.4	92.9	91.4
38 + 3 eccentric	98.6	26.7	71.4	111.6	84.4	55.2	122.0	80.2	48.6	52.7	94.1	92.8
42 + 3 eccentric	98.2	27.6	80.0	115.1	80.1	59.3	119.3	84.6	49.9	52.8	94.8	93.8
Polyethylene liner thickness (mm)												
0	62.8	10.2	46.4	71.4	57.0	26.1	95.5	63.3	37.8	41.4	76.4	74.3
6	60.4	8.0	39.2	76.0	56.7	22.1	95.3	60.6	39.0	38.0	77.1	74.5
15	72.7	8.2	39.2	73.9	57.1	22.0	97.5	60.9	40.9	38.9	77.1	74.5
Humeral NSA (°)												
125	86.5	16.4	50.8	98.0	60.8	34.4	83.3	60.7	36.1	35.9	84.1	80.1
135	62.8	10.2	46.4	71.4	57.0	26.1	95.5	63.3	37.8	41.4	76.4	74.3
145	27.3	2.6	19.3	15.9	31.4	7.2	113.1	59.7	39.0	45.2	67.5	66.0
155	3.1	0.2	2.3	0.4	4.5	0.7	118.6	55.4	40.8	36.2	53.9	49.3
Humeral stem version (°)												
+10	10.5	0.9	12.5	9.4	14.7	1.4	98.1	29.1	64.8	15.7	84.8	84.4
−10	55.5	4.4	43.4	46.0	47.6	10.7	106.2	45.1	51.1	29.5	85.8	88.4
−30	62.8	10.2	46.4	71.4	57.0	26.1	95.5	63.3	37.8	41.4	76.4	74.3
Baseplate version (°)												
0	70.0	13.6	48.0	85.1	66.8	24.7	104.8	60.6	37.7	44.2	78.4	81.6
10	66.0	6.9	45.7	55.0	60.2	15.8	96.7	54.9	36.0	32.4	82.0	77.5

*ROM*, range of motion; *NSA*, neck-shaft angle.

∗ Degrees (°).

Assessment of other implant variables revealed significant variations in impingement-free motion. Higher NSA was strongly associated with lower ROM. For example, a 125° NSA provided the greatest standard ROM (eg, 86.5° flexion, 16.4° adduction, 50.8° internal rotation, 98.0° extension, 60.8° abduction, and 34.4° external rotation), whereas a 155° NSA severely limited motion, resulting in only 3.1° of flexion and 0.7° of external rotation. Functional ADLs showed more heterogeneous responses, with elevation and reach opposite increasing with higher NSA. A −30° humeral stem version outperformed +10° or −10° settings for all standard motions such as flexion (62.8°) and extension (71.4°). Increased polyethylene liner thickness had limited impact on all motion planes. Finally, a 0° baseplate retroversion yielded higher ROM across nearly all planes compared to a 10° retroversion, with the exception of reaching back.

### Impingement points per configuration

Analysis of the most common impingement locations revealed consistent anatomic patterns across varying implant configurations (Table III). Flexion and reach opposite were almost universally limited by contact between the lesser tuberosity and the coracoid, while internal rotation consistently impinged between the polyethylene liner and the anterior glenoid. External rotation and adduction primarily encountered impingement at the posterior glenoid and inferior glenoid. Finally, extension and abduction impinged at the acromion-humerus and acromion-greater tuberosity interfaces, respectively. Notably, higher NSA shifted the primary impingement point for most standard motions to the inferior glenoid, substantially limiting motion compared to lower angles. For functional ADLs, elevation and reaching back head were frequently limited by contact between the greater tuberosity and the acromion. Reaching back and maximum reach back motions were unique in that they achieved their full ROM without any identified bony or implant impingement across all configurations.

**Table III:** Most common impingement point for standard ROM and function ROM by implant variable.

Variable	Standard ROM						Functional ROM					
	Flexion	Adduction	Internal rotation	Extension	Abduction	External rotation	Elevation	Adducted rotation	Reach opposite	Reach back head	Reach back	Max reach back
Glenosphere size (mm)												
34	LT-coracoid	Poly-PG	Poly-AG	Humerus-ACR	GT-ACR	Poly-PG	GT-ACR	Poly-PG	LT-coracoid	GT-ACR	None	None
38	LT-coracoid	Poly-IG	Poly-AG	Humerus-ACR	GT-ACR	Poly-PG	GT-ACR	Poly-PG	LT-coracoid	GT-ACR	None	None
42	LT-coracoid	Poly-PG	Poly-AG	Humerus-ACR	GT-ACR	Poly-PG	GT-ACR	Poly-PG	LT-coracoid	GT-ACR	None	None
38 + 3	LT-coracoid	Poly-IG	Poly-AG	Humerus-ACR	GT-ACR	Poly-PG	GT-ACR	Poly-PG	LT-coracoid	GT-ACR	None	None
42 + 3	LT-coracoid	Poly-IG	Poly-AG	Humerus-ACR	GT-ACR	Poly-PG	GT-ACR	Poly-PG	LT-coracoid	GT-ACR	None	None
38 Eccentric	LT-coracoid	Poly-IG	Poly-AG	Humerus-ACR	Poly-SG	Poly-PG	GT-ACR	Poly-PG	Poly-AG	Poly-PG	None	None
42 Eccentric	LT-coracoid	Poly-IG	Poly-AG	Humerus-ACR	Poly-SG	Poly-PG	GT-ACR	Poly-PG	LT-coracoid	GT-ACR	None	None
38 + 3 Eccentric	LT-coracoid	Poly-IG	Poly-AG	Humerus-ACR	GT-ACR	Poly-PG	GT-ACR	Poly-PG	LT-coracoid	Poly-PG	None	None
42 + 3 Eccentric	LT-coracoid	Poly-IG	Poly-AG	Humerus-ACR	GT-ACR	Poly-PG	GT-ACR	Poly-PG	LT-coracoid	GT-ACR	None	None
Polyethylene liner thickness (mm)												
0	LT-coracoid	Poly-IG	Poly-AG	Humerus-ACR	GT-ACR	Poly-PG	GT-ACR	Poly-PG	LT-coracoid	GT-ACR	None	None
6	LT-coracoid	Poly-PG	Poly-AG	Humerus-ACR	GT-ACR	Poly-PG	GT-ACR	Poly-PG	LT-coracoid	GT-ACR	None	None
15	LT-coracoid	Poly-IG	Poly-AG	Humerus-ACR	GT-ACR	Poly-PG	GT-ACR	Poly-PG	Poly-AG	GT-ACR	None	None
Humeral NSA (°)												
125	LT-coracoid	Poly-PG	Poly-AG	Humerus-ACR	GT-ACR	Poly-PG	GT-ACR	Poly-PG	LT-coracoid	GT-ACR	None	None
135	LT-coracoid	Poly-IG	Poly-AG	Humerus-ACR	GT-ACR	Poly-PG	GT-ACR	Poly-PG	LT-coracoid	GT-ACR	None	None
145	Poly-IG	Poly-IG	Poly-IG	Poly-IG	Poly-IG	Poly-PG	GT-ACR	Poly-PG	LT-coracoid	GT-ACR	None	None
155	Poly-IG	Poly-IG	Poly-IG	Poly-IG	Poly-IG	Poly-IG	GT-ACR	Poly-PG	LT-coracoid	Poly-PG	None	None
Humeral stem version (°)												
+10	Poly-PG	Poly-PG	Poly-PG	Poly-PG	Poly-PG	Poly-PG	Poly-PG	Poly-PG	None	Poly-PG	None	None
−10	LT-coracoid	Poly-PG	Poly-AG	Poly-PG	GT-ACR	Poly-PG	GT-ACR	Poly-PG	LT-coracoid	Poly-PG	None	None
−30	LT-coracoid	Poly-IG	Poly-AG	Humerus-ACR	GT-ACR	Poly-PG	GT-ACR	Poly-PG	LT-coracoid	GT-ACR	None	None
Baseplate version (°)												
0	LT-coracoid	Poly-PG	Poly-AG	Humerus-ACR	GT-ACR	Poly-PG	GT-ACR	Poly-PG	LT-coracoid	Poly-PG	None	None
10	LT-coracoid	Poly-PG	Poly-AG	Poly-PG	GT-ACR	Poly-PG	GT-ACR	Poly-PG	LT-coracoid	Poly-PG	None	None

*GT*, greater tuberosity; *LT*, lesser tuberosity; *ACR*, acromion; *SG*, superior glenoid; *IG*, inferior glenoid; *AG*, anterior glenoid; *PG*, posterior glenoid; *ROM*, range of motion; *NSA*, neck-shaft angle.

### Multivariable linear regression of optimal implant placement

Multivariable linear regression used standardized beta coefficients to evaluate the strength of association of each implant parameter for achieving maximal ROM (Table IV). Overall, the NSA and glenosphere size were identified as the strongest independent predictors of maximal ROM across multiple planes. A humeral NSA of 125° had the highest magnitude of effect for improving standard motions, particularly flexion (beta = 0.93, *P* < .05), external rotation (beta = 1.10, *P* < .05), and adduction (beta = 1.21, *P* < .05). NSA of 155°, meanwhile, was significantly associated with decreased ROM in nearly all motions. Glenosphere size was a significant determinant of ROM, with the 42-mm configuration associated with significantly improved motion across nearly all planes, whereas the 34-mm configuration was associated with significantly reduced ROM across nearly all motions. Furthermore, both eccentric glenosphere positioning and the use of +3 mm lateralization were significant independent predictors with increased ROM across nearly all planes (*P* < .05). While humeral stem version influenced several planes, with the −30° version showing strong positive associations with extension and external rotation, baseplate retroversion and polyethylene liner thickness generally showed fewer significant associations across the full ROM parameters.

**Table IV:** Multivariable linear regression showing standardized beta coefficients for each ROM by implant variable.

Variable	Standard ROM						Functional ROM					
	Flexion	Adduction	Internal rotation	Extension	Abduction	External rotation	Elevation	Adducted rotation	Reach opposite	Reach back head	Reach back	Max reach back
Glenosphere size (mm)												
34	−0.37∗	−0.84∗	−0.60∗	−0.35∗	−0.27	−0.84∗	−0.19	−0.50∗	−0.23	−0.18	−0.41∗	−0.47∗
38	0.09	0.22∗	0.21∗	0	0.08	0.16∗	0.08	0.13	0.15∗	0.27∗	−0.03	−0.02
42	0.25∗	0.49∗	0.58∗	0.31∗	0.16∗	0.51∗	0.08	0.38∗	0.29∗	0.39∗	0.27∗	0.22∗
Lateralization (mm)												
0	−0.05	−0.01	0.21∗	−0.02	−0.02	−0.01	−0.06	0.04	−0.04	0.16∗	−0.05	−0.07
3	0.30∗	0.51∗	0.38∗	0.25∗	0.20∗	0.46∗	0.18∗	0.33∗	0.43∗	0.42∗	0.19∗	0.17∗
Eccentricity												
Concentric	−0.08	−0.25∗	−0.01	−0.1	−0.16∗	−0.22∗	−0.12	−0.07	0.04	0.15∗	−0.06	−0.09
Eccentric	0.35∗	0.81∗	0.66∗	0.35∗	0.37∗	0.72∗	0.26∗	0.47∗	0.33∗	0.43∗	0.21∗	0.19∗
Polyethylene liner thickness (mm)												
0	0.18	0.22	0.22	0.22	0.29	0.22	0.1	0.09	0.11	0.27	0.14	0.08
6	0.18	0.22	0.22	0.22	0.21	0.22	0.01	0.09	0.16	0.28	0.17	0.09
15	0.43∗	0.24	0.22	0.18	0.23	0.22	0.07	0.11	0.27	0.33∗	0.17	0.09
Humeral NSA (°)												
125	0.93∗	1.21∗	0.87∗	0.99∗	0.67∗	1.10∗	−0.30∗	0.2	0.14	0.17	0.50∗	0.43∗
135	0.40∗	0.26∗	0.48∗	0.59∗	0.64∗	0.43∗	0.04	0.19	0.22	0.24	0.28	0.27
145	−0.26∗	−0.35∗	−0.16	−0.50∗	−0.09	−0.37∗	0.33∗	0.14	0.27	0.51∗	0.04	0.06
155	−0.75∗	−0.60∗	−0.71∗	−0.75∗	−0.77∗	−0.71∗	0.44∗	−0.06	0.37∗	0.13	−0.34∗	−0.33∗
Humeral stem version (°)												
−30	0.53∗	0.72∗	0.34∗	0.76∗	0.59∗	0.79∗	0.04	0.78∗	−0.34∗	0.68∗	−0.06	−0.16
−10	0.42∗	0.12	0.45∗	0.17	0.28	0.1	0.16	0.08	0.06	0.26∗	0.22	0.26
10	−0.52∗	−0.45∗	−0.42∗	−0.56∗	−0.54∗	−0.46∗	0.07	−0.65∗	0.55∗	−0.45∗	0.19	0.15
Baseplate version (°)												
0	0.33	0.65∗	0.38	0.33	0.3	0.26	0.2	0.06	0.18	0.44∗	0.07	0.18
10	0.31	0.05	0.42∗	−0.1	0.2	−0.13	0.06	−0.11	0.16	0.01	0.28	0.17

*ROM*, range of motion; *NSA*, neck-shaft angle.

∗ *P* value <.05.

## Discussion

Advances in technology have expanded the use of CT-based 3D simulation models to help optimize implant positioning and improve patient outcomes. This study adds to this growing field by using a novel 3D computer model that incorporates both glenohumeral and scapulothoracic motion. Because scapulothoracic motion plays a major role in both normal and pathologic shoulder function, its inclusion makes the model more realistic and strengthens the clinical relevance of its findings.^4^^,^^28^^,^^36^^,^^44^ The most important finding of this study was that rTSA implant selection and component positioning was shown to influence impingement-free ROM. Our findings support the conclusion that implant positioning significantly influences impingement-free ROM following rTSA. Specifically, glenosphere size and positioning, as well as NSA, were the 2 most important predictors of both standard and functional ROM. In contrast, polyethylene liner thickness and baseplate retroversion did not have consistent impacts on ROM. These results reinforce the growing body of literature suggesting that patient-specific 3D planning is a critical tool to improve postoperative functional outcomes.

This study showed that glenosphere size significantly influenced ROM across both standard and functional planes of motion. Specifically, the 42-mm glenosphere was significantly associated with improvements in all motions compared to 34-mm size. This is consistent with prior literature regarding the impact of glenosphere parameters, both in virtual modeling and retrospective analysis.^7^^,^^30^^,^^33^^,^^39^^,^^41^ Increasing glenosphere size can delay impingement by increasing the arc of motion. In other words, a larger-radius glenosphere moves the humeral component farther from potential impingement sites on the scapula.^9^^,^^21^^,^^41^

Similarly, eccentricity and positive lateralization had significant benefits across all standard and functional motion planes. The strongest impacts were on adduction, internal rotation, and external rotation for eccentricity, and adduction, external rotation, and reach opposite for lateralization. Arenas-Miquelez et al^5^ and Werner et al^41^ had similar findings in their modeling studies. Both eccentricity and lateralization work in similar mechanisms by shifting the center of rotation inferiorly and laterally, respectively. Inferior eccentricity allows for improved adduction angles before contacting the inferior scapular neck. Lateral movement increases the moment arm of the deltoid and increases distance between the humeral component and scapular neck, similarly to an increased glenosphere size.^6^^,^^9^^,^^11^

The humeral NSA was found to be one of the most critical determinants of motion in this model, with lower angles providing greater ROM across all standard planes and higher angles resulting in nearly total loss of ROM. This is supported by the impingement data, showing higher NSA configurations led to nearly immediate contact between the polyethylene liner and the inferior glenoid at under 5° in any standard direction. These findings are consistent with prior literature, with the exception that lower NSAs have been associated with decreased abduction.^42^ This may be due to differences in default implant parameters, such as glenosphere size and lateralization, though this finding requires future research.^31^^,^^38^ The simulation of functional ADLs in this study provides additional clinical context. While standard motions are often the focus of clinical assessment, functional tasks like reach back head or reach back are vital to patient satisfaction. Interestingly, while NSA displayed a very clear pattern of improving standard motion planes, higher angles gave slight advantages in elevation and reach opposite. This may be because distalizing the implant with a higher NSA increases the acromiohumeral space, allowing greater elevation and reach opposite before impingement occurs. These findings underscore the trade-offs surgeons must navigate when selecting an NSA based on a patient's specific functional goals. Furthermore, clinical application of these findings requires careful consideration of soft tissue constraints not captured in this bony simulation model.

Another finding from the functional ADLs data was that reaching back and maximum reaching back motions achieved their maximal ROM without any impingement across all implant configurations. This stands in contrast to standard motions like flexion or abduction that were consistently limited by the scapular body and glenoid. This finding may be because this is a complex multiplanar motion that uses a sequence of movements that can navigate typical impingement zones.^10^^,^^33^ Alternatively, impingement from these movements may be more closely tied to soft tissue impingement rather than bony impingement.^20^ With this knowledge, the restoration of reaching back may depend more heavily on postoperative rehabilitation and management of soft tissue constraints.

### Limitations

This study has several limitations. First, given the recency of these surgeries, we were not able to correlate the ROM findings with actual postoperative patient outcomes and functional scores. Patients may thus postoperatively exhibit different compensatory kinematic patterns to what was modeled. Future work would correlate postoperative follow-up to the modeling output seen in this study. Second, the 3D simulation focused primarily on bone-on-bone or implant-on-bone impingement and thus does not account for the influence of soft tissue structures such as the deltoid or remaining rotator cuff musculature. This limitation is particularly relevant when interpreting the functional results, as these complex motions may be substantially limited by soft tissue constraints in clinical practice. Additionally, our simulations identify implant configurations that maximize impingement-free ROM but do not assess the risks of soft tissue overtensioning. Third, during the 3D simulation planning process, osteophytes were not removed before the simulations. While relative trends between implant configurations would likely remain consistent, osteophyte removal would influence impingement points and ROM values and could be an area of future study. Fourth, results were generated using 1 3D planning software and thus specific implant configurations may vary if analyzed with different 3D planning platforms. Finally, CORIOGRAPH MODELER simulations are evaluations of healthy motion that can be achieved before compensatory scapula motion is triggered. rTSA patients present modified movement patterns and using a healthy model simulator can impact the interpretation of the predicted ROM.

## Conclusion

Using a 3D simulation model incorporating scapulothoracic motion, rTSA implant positioning was shown to influence impingement-free ROM. Larger and eccentric glenospheres with increased lateralization consistently improved ROM, while higher NSAs limited motion across multiple planes. NSA and glenosphere size were the strongest independent predictors of maximal ROM.

## Disclaimers:

Funding: No funding was disclosed by the authors.

Conflicts of interest: Eoghan T. Hurley is on the editorial board for Arthroscopy, European Society for Surgery of the Shoulder and Elbow, and Journal of Shoulder and Elbow Surgery. Alessandro Navacchia is an employee of Smith + Nephew. Alessandro Navacchia is an employee of Smith & Nephew. Michael Moverman reports professional activities at DJO, LLC. Jay M. Levin holds stock with Zimmer Biomet Holdings, Inc. and Stryker. Christopher S. Klifto is a consultant for Smith + Nephew, Acumed, LLC, Restore3d, and Stryker and holds stocks with GE Healthcare, Johnson & Johnson, Merck, and Pfizer. Any additional authors, their immediate families, and any research foundations with which they are affiliated have not received any financial payments or other benefits from any commercial entity related to the subject of this article.
